# mmp-9 mRNA Expression and Bridging Fibrosis Progression in Toxic Liver Injury

**DOI:** 10.32607/actanaturae.17856

**Published:** 2023

**Authors:** E. I. Lebedeva, A. S. Babenka, A. T. Shchastniy

**Affiliations:** Vitebsk State Order of Peoples’ Friendship Medical University, Vitebsk, 210009 Republic of Belarus; Belarussian State Medical University, Minsk, 220116 Republic of Belarus

**Keywords:** rats, liver, mmp-9 mRNA, immunohistochemistry, FAP+, α-SMA+, CD45+ cells

## Abstract

Developing liver disease treatments, in which fibrosis is a key pathogenetic link, still remains an urgent problem in hepatology. In the present study, the level of *mmp-9 *mRNA expression and the number of FAP+, α-SMA+, CD45+ cells were analyzed at nine time points of fibrosis and cirrhosis. It was found that in the case of liver fibrosis, the choice of the optimal reference gene depended on the stage of fibrogenesis. When studying the specific stages rather than the entire process in a long-term experiment, it was shown that choosing an optimal reference gene has to be done additionally. In this case, the *mmp-9 *mRNA expression level should be considered as a marker of liver fibrosis initiation and development but not as that of cirrhosis progression. In the liver, two morphologically heterogeneous populations of myofibroblasts were simultaneously identified as able to synthesize various types of immunohistochemical markers. It was found that the FAP+ cells were the main contributor to the development of portal fibrosis and the initial stages of bridging fibrosis. In the selected experimental model, fibrosis initiation and the development stages preceding parenchyma restructuring were accompanied by a low level of inflammation.

## INTRODUCTION

The MMP-9 protein, also known as type IV collagenase (gelatinase B), belongs to a large family of matrix zinc-dependent proteinases (MMPs). Almost all members of this family play an important role in liver regeneration and in the control over the number of extracellular matrix proteins. They also participate in fibrosis, cirrhosis, carcinogenesis, and other processes [1]. Many years of studying the molecular mechanisms of liver fibrosis development have made it clear that the MMP-2 and MMP-9 proteins are involved in this pathological process at almost all stages and perform a key function in its progression [2, 3]. At the same time, the number of MMPs in blood plasma is a marker of fibrosis and some therapeutic approaches are aimed at MMP-9 as a specific target [4, 5, 6, 7].

Some researchers have noted an increase in the expression of the MMP-9 protein and corresponding mRNA in the presence of progressing liver fibrosis, while the etiological factors causing fibrosis are not that important. An increase in MMP-9 expression has been observed in toxic damage to the liver, as well as in the presence of viral hepatitis [8, 9]. As fibrosis develops, an increase in the specific amount of connective tissue occurs and, in some cases, a correlation between this process with an increase in the level of* mmp-9 *mRNA has been reported. In normal conditions, it is metalloproteinase that stands responsible for the degradation of connective tissue, collagen renewal, and maintenance of the optimal level of extracellular matrix proteins [10, 11]. However, the relationship between an increased level of *mmp-9 *mRNA and the progression of liver fibrosis has yet to be studied [1, 2, 3]. In addition, existing experimental animal models have been designed to investigate specific key positions that are rather far apart from each other (norm, fibrosis and cirrhosis, or fibrogenesis) and they are usually studied within a relatively short time period. These limitations may cause the models to miss the important details relative to *mmp-9 *level dynamics [8, 9, 10, 11].

Stellate cells (HSCs) are considered to be the main cell population synthesizing the intercellular substance in liver pathologies. In the scientific literature, they are known under different names, such as fat-accumulating cells, lipocytes, perisinusoid cells, hepatic stellate cells, Ito cells, and pericytes [12, 13, 14, 15]. Under physiological conditions, HSCs are localized in the perisinusoidal space; they regulate the blood flow in the sinusoids, functioning as pericytes, and possess low proliferative capacity and the ability to secrete collagens [13, 15]. Liver lesions of predominantly viral and toxic etiology stimulate HSC activation and transdifferentiation into a myofibroblastic phenotype, with an overexpression of α-SMA [12, 14, 15, 16]. The activation and transdifferentiation processes are not yet fully understood, which is why an effective antifibrotic therapy has not yet been developed. The source of resting and activated HSCs has also not been established yet. Their pool is assumed to be replenished by bone marrow cells, but it cannot be ruled out that this is a self-sustaining cell population [12, 16, 17, 18].

These are resident portal fibroblasts (PFs) that are considered to be the source of myofibroblasts in cholestatic liver diseases [13], but their role in the development of cholestatic fibrosis remains debatable. In the studies using Col-GFP and Mdr2-/-mice, PFs served as the source of myofibroblasts at the initial stages of cholestatic fibrogenesis, whose further progression led to HSC transdifferentiation into a fibrogenic phenotype [16]. Other authors note that cholestatic fibrosis is accompanied by simultaneous PF and HSC activation [16, 17, 18].

For the purposes of this study, we had hypothesized that the increase in the level of *mmp-9 *mRNA might be associated with the rate of connective tissue formation in fibrogenesis; so, the objective of our investigation was to probe for new data on the level of* mmp-9 *mRNA expression and fibrogenic cell population at different stages of toxic liver fibrosis.

## EXPERIMENT

The design of the experiment was approved at a meeting of the Commission on Bioethics and Humane Treatment of Laboratory Animals of Vitebsk State Order of Peoples’ Friendship Medical University (Minutes No. 6 of 01/03/2019) and involved mature male Wistar rats weighing 190–210 g. Liver fibrosis and cirrhosis were modeled by chronic intoxication with thioacetamide (TAA; Acros Organics). A freshly prepared TAA solution was administered intragastrically through a tube at a dose of 200 mg/kg of body weight twice a week for 17 weeks. The rats comprising the control group (*n *= 12) received TAA-free water in the same volume. The animals were randomized into 8 groups (*n *= 12 in each) depending on TAA exposure duration: 3 weeks (Group 1), 5 weeks (Group 2), 7 weeks (Group 3), 9 weeks (Group 4), 11 weeks (Group 5), 13 weeks (Group 6), 15 weeks (Group 7), and 17 weeks (Group 8).

**Applied histological and morphometric methods**

After guillotine decapitation under short-term ether anesthesia, samples of 5–10 mm in diameter were taken from the large left lobe of rat liver to be fixed for 24 h in a 10% neutral formalin phosphate buffer solution (Biovitrum, Russia). The fixed material was embedded in paraffin using an STP-120 spin tissue processor (Thermo Fisher Scientific, Germany) and an EC350 modular paraffin embedding center (Thermo Fisher Scientific). From each animal, one preparation was obtained for each staining method and using an HM340E rotary microtome (MICROM, Laborgerate GmbH, Germany). An average of 3–4 sections with a thickness of 4 μM were prepared and placed on glass slides. To get the overview histological preparations, the liver sections were stained with hematoxylin and eosin; and to identify connective tissue, they were stained as per Mallory in an HMS70 staining machine (Thermo Fisher Scientific) [19].

Immunohistochemical study was performed on paraffin sections [20]. Such markers as rabbit polyclonal antibodies FAP (FAP-alpha, FAP prolyl endopeptidase, dilution 1 : 100) were applied for activated PFs; activated HSC - mouse monoclonal antibodies (alpha-SMA, ASTA2, dilution 1 : 1000) for α-SMA, and hematopoietic stem cells for rabbit polyclonal antibodies (CD45, dilution 1 : 200). The antibodies were manufactured by Wuman Elabscience Biotechnology Incorporated Company, catalog number E-AB-32870 (FAP), E-AB-22155 (α-SMA), E-AB-16319 (CD45). For investigation purposes, we also employed a 2-step Plus Poly-HRP Anti Rabbit/Mouse IgG Detection System with the DAB Solution kit; Retrieve-All Antigen (Unmasking System Basic), Antibody Dilution Buffer (BioLegend), Tween-20 (Glentham Life Sciences), and PBS (Melford). For better orientation in the preparation and correct identification of the cells containing the desired antigens, the sections were counterstained with Mayer’s hematoxylin for 1 min. For an objective interpretation of the results for each group in the study, both positive and negative controls were utilized: immunohistochemical staining was assessed as positive only in the absence of staining in the negative control and, conversely, as negative when staining was detected in the positive control.

**Morphometric analysis**

Histological preparations were examined using the ImageScope Color and cellSens Standard software. The connective tissue area was determined as a percentage of the total section area [21]. The measurements were carried out using an OLYMPUS XC30 digital camera (Japan) based on an OLYMPUS BX51 microscope (Japan) of 20× magnification to take microphotographs of the random vision fields (at least 3 in each histological section) of the liver preparations. The number of FAP+-positive cells (FAP+ cells), α-SMA-positive cells (α-SMA+ cells), and CD45- positive cells (CD45+ cells) was counted in the three vision fields of each histological section at a 40× magnification. The degree of fibrosis was assessed using the semi-quantitative scale devised by K.G. Ishak (*Table 1*) [22, 23].

**Table 1:** Stages of liver fibrosis as scaled by K.G. Ishak

Scaled liver fibrosis stages	Morphological characteristics of fibrosis severity
F0	No fibrosis
F1	Fibrous enlargement of the portal zones with and without short fibrous septa
F2	Fibrous expansion of most portal zones with and without short fibrous septa
F3	Fibrous expansion of most portal zones with single bridging portoportal septa
F4	Fibrous expansion of most portal zones with pronounced bridging portoportal and portocentral septa
F5	Numerous bridge-like septa with single nodules (incomplete cirrhosis)
F6	Cirrhosis

***mmp-9*-gene mRNA relative level estimation**

To investigate *mmp-9 *mRNA, the liver samples were placed in cryovials and then in liquid nitrogen for storage before the start of a total RNA isolation procedure. The total RNA fraction was isolated as per the manufacturer’s instructions for the ArtRNA MiniSpin kit (ArtBioTech, Belarus). cDNA was synthesized using oligo(dT) primers and the ArtMMLV Total kit (ArtBioTech) according to the manufacturer’s instructions. In each reaction, 200 ng of the total RNA fraction was used. Oligonucleotide primers and real-time polymerase chain reaction (RT-PCR) probes were selected using the free online application Primer3 v. 0.4.0 (http://bioinfo.ut.ee/primer3-0.4.0/). *Hes1, sdha,* and *hprt *were chosen as reference gene candidates. The oligonucleotide sequences are presented in *Table 2*.

Real-time PCR (RT-PCR) was performed using reagents manufactured by Primetech, Belarus. The final volume of the reaction mixture was 25 µl and contained all the necessary components in the following concentrations: 2 mM of magnesium chloride; 0.1 mM of a mixture of deoxynucleotide triphosphates; 500 nM of oligonucleotides, including a real-time PCR probe; and 1.25 units of thermostable Taq-DNA polymerase in the appropriate buffer solution. Thermal cycling included one 2-min cycle at 95°C followed by 40 5-second cycles at 95°C and a 45-second cycle at 60°C. FAM channel detection was performed after each cycle. To perform the PCR, the CFX96 Touch Real-Time PCR Detection System was employed (BioRad, USA). The efficiency of the reactions was determined using the standard curve method and series of dilutions of concentrated cDNA samples. RT-PCR of each sample was carried out in three repetitions. In each experimental and in the control group, each of the 12 samples was analyzed separately to achieve the highest reliability and account for the intragroup variation and phenotypic heterogeneity of the gene expression level.

**Table 2:** Oligonucleotide primers and fluorescently labeled markers used in the study

Oligonucleotide	Nucleotide sequence, 5’ → 3’	Marker, 5’	Marker, 3’
mmp-9F	CTACTCGAGCCGACGTCAC		
mmp-9R	AGAGTACTGCTTGCCCAGGA		
mmp-9P	GATGTGCGTCTTCCCCTTCG	FAM	BHQ1
hes1F	GAAAGATAGCTCCCGGCATT		
hes1R	CGGAGGTGCTTCACTGTCAT		
hes1P	CCAAGCTGGAGAAGGCAGACA	FAM	BHQ1
hprtF	GGACAGGACTGAAAGACTTGCT		
hprtR	ACAGAGGGCCACAATGTGAT		
hprtP	CATGAAGGAGATGGGAGGCC	FAM	BHQ1
sdhaF	CCCACAGGTATCTATGGTGCT		
sdhaR	TTGGCTGTTGATGAGAATGC		
sdhaP	CATCACAGAAGGGTGCCGTG	FAM	BHQ1

**Statistical analysis**

The obtained results were processed in Statistica 10.0 (StatSoft, Inc.) and Microsoft Office Excel (Microsoft Corp.). For each sample, the normality of the frequency distribution of each feature was determined. Since the samples were not small (*n *= 60>50), the tests were carried out with application of the Lilliefors criterion. The data were presented as arithmetic means (M) and corresponding confidence intervals (95% CI), a median, and the 15^th^ and 85^th^ percentile values (Me (15%; 85%)). The level of statistical significance of the differences in the studied characteristics in the groups with normal data distribution was assessed using the Student’s t-test; if the samples differed from the normal distribution, the Mann–Whitney U-test was used. For clarity, the results of the statistical analysis were presented as graphs of a one- and two-factor parametric variance analysis that was permissible to apply, since all groups had the same number of studied characteristics [24].

## RESULTS

**Pathological analysis of rat liver**

**Fig. 1:**
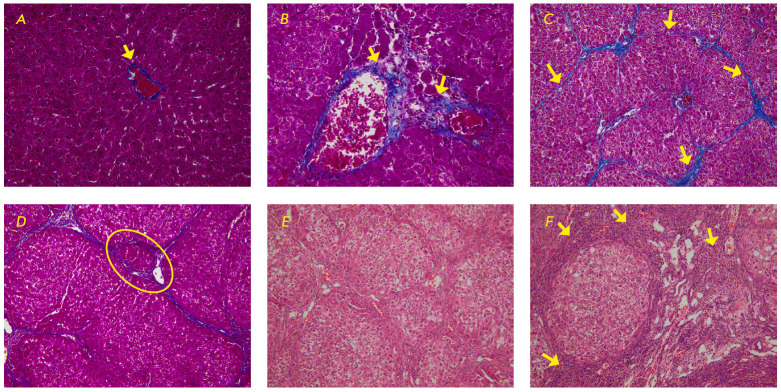
Fragments of the liver of the control group rats: (*A*) at 3 weeks, (*B*) at 7 weeks, (*C*) at 9 weeks, (*D*) at 13 weeks, (*E*) at 17 weeks, (*F*) before the beginning of the experiment. Mallory staining ×40 (*A*, *B*); ×20 (*C*, *D*). Hematoxylin-eosin staining ×20 (*E*, *F*). (*A*) – a small amount of connective tissue in the central vein region (marked with an arrow); (*B*) – connective tissue in the portal zone (marked with arrows); (*C*) – connective tissue septa between portal zones (marked with arrows); (*D*) – neoformed false hepatic lobule (marked with an oval frame); (*E*) – neoformed false hepatic lobules; (*F*) – pronounced liver destruction with clearly visualized lymphoid-histiocytic infiltrate cells (marked with arrows)

**Fig. 2:**
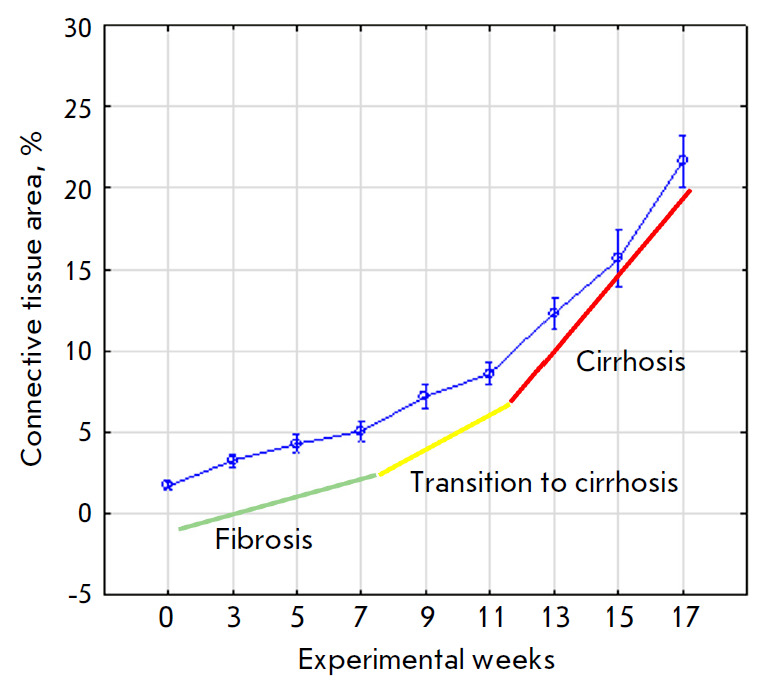
Changes in the connective tissue area at different stages of the study

In the animals of the intact group, a small amount of connective tissue was found around the interlobular vessels and bile ducts of the portal zones, as well as the central and collecting veins (F0, *Fig. 1A*). It is noteworthy that as liver fibrosis progressed, the rate of connective tissue growth varied (*Fig. 2*).

By the 3^rd^ week into the experiment, a moderate formation of fibrous connective tissue was observed in the portal zones (F1, *Fig. 1B*). In the 5^th^ week, the formation of fibrous tissue slowed down, but at the same time it was simultaneously detected both in the portal zones and in the parenchyma (bridging fibrosis, F2/F3). By the 7^th^ week, the intensity of connective tissue synthesis remained almost at the same level as in the 5^th^ one (F3/F4, *Fig. 1C*). At the stage of transition from fibrosis to cirrhosis, an increased formation of connective tissue similar to that in the 3^rd^ week of the experiment was observed, again. In nine weeks, in the portal zones, the formation of false hepatic lobules occurred, a morphological criterion for initial fibrosis to cirrhosis transition (F4/F5, *Fig. 1D*). In the period from the 11th to 17^th^ week, connective-tissue proliferation reached its maximum value (F6, *Fig. 1 E,F*).

In the liver of the intact animals, cells of the lymphoid- histiocytic infiltrate were practically absent, which was an indication of either extremely low inflammation severity or its complete absence. Contrary to popular belief that the inflammation level increases as fibrosis develops, by the 3^rd^ week and then at the 5 and 7^th^ weeks, we did not observe morphologically significant inflammation foci. That was the clue that, before the start of parenchyma restructuring, fibrosis initiation and development were accompanied in this toxic model by a low level of inflammation. Starting from the 9th week at the stage of active transition of fibrosis to cirrhosis, diffuse inflammation foci were observed in the connective tissue septa and the portal zones. By the 11th week (stage of incomplete cirrhosis), the level of inflammation was assessed as moderate; so, the number of lymphoid-histiocytic cells increased. From the 13 to 17^th^ week at the stage of advanced cirrhosis, the level of inflammation rapidly increased, to be regarded as high (*Fig. 1 E,F*).

**Changes in the number of cells expressing FAP, α-SMA, and CD45 markers**

**Fig. 3:**
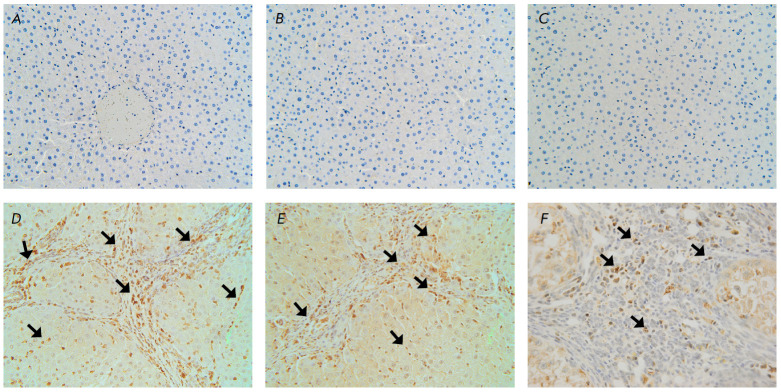
Fragments of the liver of the control group rats: (*A, B, C*) at 15 weeks, (*D, E*) at 17 weeks, (*F*) before the beginning of the experiment. Immunohistochemical staining (restained with Mayer’s hematoxylin): for FAP (*A*, *D*); for α-SMA (*B*, *E*); for CD45 (*C*, *F*). Magnification ×40: (*A*) no FAP+-cells are found; (*B*) no α-SMA+-cells are found in the sinusoids; (*C*) no CD45+-cells are found in the parenchyma; (*D*) α-SMA+-cells (marked with arrows); (*E*) FAP+-cells (marked with arrows); (*F*) CD45+-cells in the connective tissue (marked with arrows)

The cells synthesizing the FAP+ marker were absent in the livers of the intact animals (*Fig. 3A*). No α-SMA^+^ cells were observed in the sinusoids (*Fig. 3B*), but in some cases they were detected in the walls of the interlobular arteries, as well as in the interlobular and sublobular veins. CD45+ cells were almost never found in the lumens of the blood vessels and sinusoids; however, they were not visualized in the parenchyma, either (*Fig. 3C*).

**Fig. 4:**
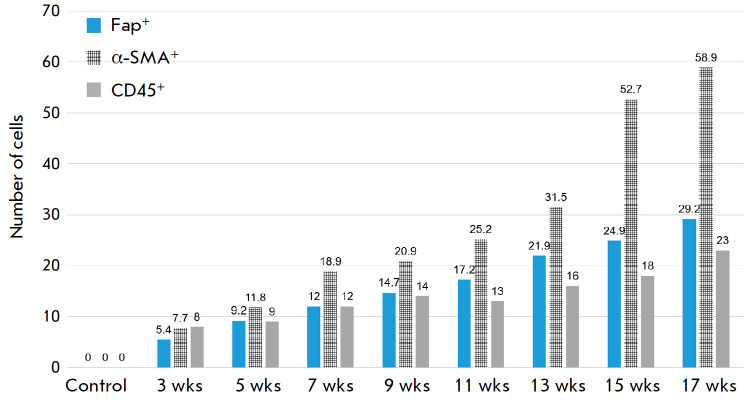
Changes in the number of FAP+-, α-SMA+-, and CD45+- cells at different stages of the study

Starting from week three, the number of cells bearing these markers increased, and the number of α-SMA^+^-, CD45^+^ cells began to exceed that of FAP^+^- cells (*Fig. 4*). At the 5^th^ week, the number of cells carrying the target markers increased while the gap between the FAP^+^ and α-SMA^+^ cells narrowed, and the increase in CD45^+^ cells became minimal. Then, the situation with FAP^+^- and α-SMA^+^-cells repeated itself in the 7^th^ and 9th weeks. In the 7^th^ week, the increase in the number of α-SMA^+^ cells, in percentage terms, was more pronounced, while in the 9th week the gap between the number of FAP^+^ and α-SMA^+^ cells had narrowed again. The number of CD45^+^ cells grew as well, but, as fibrosis progressed, its rate dropped, making this parameter a minor one in growth-rate terms. From the eleventh to the thirteenth weeks, while transition from fibrosis to cirrhosis was under way, the gap in the growth rate of FAP^+^- and α-SMA^+^-cells again appeared, in addition to which a slight decrease in the number of CD45^+^-cells was recorded. Despite the observed increase, their share at the 15^th^ and 17^th^ weeks remained the lowest when compared to the FAP^+^- and α-SMA^+^-cells, while the number of α-SMA^+^ cells had increased rapidly. At all time, a statistically significant strong correlation was found between the area of connective tissue and the number of FAP^+^-, α-SMA^+^-, and CD45^+^-cells.

In the histological preparations, rounded α-SMA+ cells were observed in the sinusoids and necrotizing foci before the onset of the fibrosis to cirrhosis transition (9th week). From the 11th to 17^th^ week, they were located both in the sinusoids and in the connective tissue septa (*Fig. 3D*). At the first stage of the experiment, rounded FAP+ cells were localized around the interlobular vessels and near the interlobular bile ducts of the portal zones and from the 7^th^ week they were detected in the connective tissue septa and sinusoids (*Fig. 3D*).

Using Mallory’s staining method, we observed the directed growth of fibrous connective tissue fibers with FAP+ cells from two portal zones through the liver parenchyma towards each other, predetermining the path for bridging fibrosis that is a formation of pathological tissue and connective tissue bridges. The CD45+ cells were diffusely localized among other cells of the lymphoid-histiocytic infiltrate in the connective tissue septa and portal zones, as well as in the blood vessel lumens (*Fig. 3F*). Less commonly, they were detected in the sinusoids of false hepatic lobules.

***mmp*-9 mRNA expression level**

**Fig. 5:**
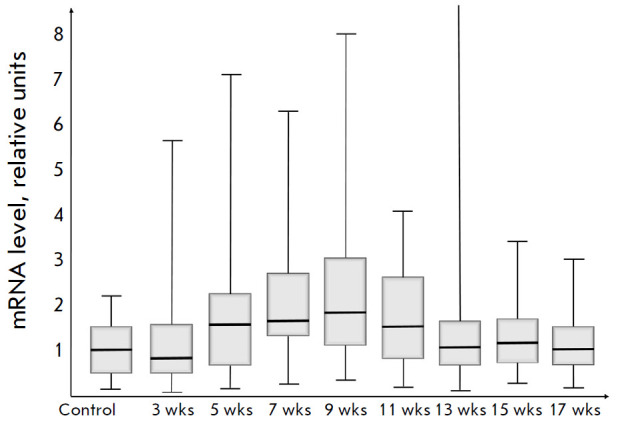
Relative level of *mmp-9 *mRNA

To normalize the RT-PCR data, the *hes1 *gene was chosen as a reference one, since its expression level proved the most stable throughout the experiment. The use of *hprt1 *and *sdha *as reference genes was considered inappropriate due to the high variability of their mRNA levels. RT-PCR efficiency for the target (*mmp-9*) and the reference gene differed by less than 1% [25]; so, the relative mRNA level was assessed using the standard Livak and Schmittgen’s method [26]. The data on a normalized level of *mmp-9 *mRNA expression are shown in *Fig. 5*.

The analysis included all values obtained within the study, not excluding the “outliers” with a low level of* mmp-9 *mRNA observed at the control point (intact rats). It is noteworthy that by the 3^rd^ week into the experiment, in the presence of developing fibrosis, the relative *mmp-9 *level did not increase and even slightly decreased when compared to the control value. At the same time, it increased over a relatively short interval between the 5^th^ and 9th weeks to subsequently drop to its initial level. In the presence of increased* mmp-9 *mRNA expression, the fibrosis to cirrhosis transition occurred. Starting from the 11th week, the level of *mmp-9 *mRNA began to drop and, as a result, already from the 13th to 17^th^ weeks, it had matched the initial one at the control point.

**Fig. 6:**
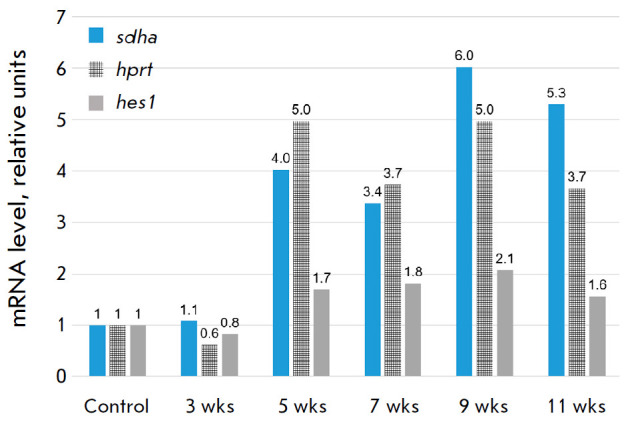
Relative *mmp-9 *mRNA level for different genes used to normalize RT-PCR data

It is important to note that an increase in the level of *mmp-9 *mRNA while fibrogenesis is underway has been noted in many studies performed on laboratory animals [27, 28]. However, researchers rarely insist on both a detailed analysis of all fibrosis stages and on choosing an appropriate reference gene for RT-PCR data normalization. *Figure 6* shows our data normalized using the two other reference genes (*hprt1, sdha*), whose range of Ct values in the experiment was higher than that of the target gene (*mmp-9*). In other words, *Fig. 6* exemplifies an inadequate use of reference genes for RT- PCR data normalization; e.g., when applying *hprt1*, an average increase in the *mmp-9 *mRNA level was recorded that also decreased in the 3^rd^ week. Given that relative characteristics vary widely, we believe the accuracy of such measurements can be sufficient only for a small number of experimental control points; so, we do not recommend using *hprt1 *for a detailed analysis of fibrosis stages. Applying *sdha*, on the other hand, made it impossible to register a drop in the *mmp*-9 level at the 3^rd^ week and at the beginning of the 11th week. Here, it is noteworthy that the choice of an optimal reference gene depends on the stage of fibrosis. In a detailed study of its specific stages, rather than that of the entire process in a long-term experiment, one should make sure to additionally select an optimal reference gene [19].

## DISCUSSIONS

In the framework of this study, we did not assess the level of the MMP-9 protein and cannot state which cells synthesize it, for this will be the subject of further research. In our study, MMP-9 was shown to be secreted in the liver mainly by Kupffer cells (resident macrophages) [29, 30]. MMP-9 activates latent TGFβ (transforming growth factor beta) and, thus, promotes HSC transdifferentiation into the myofibroblastic phenotype and further progression of liver fibrosis [31, 32, 33]. At the same time, Atta H. et al. note that MMP-9 can promote apoptosis of transformed HSCs at a low level of TIMP1 (tissue inhibitor of matrix metalloproteinases) and Kupffer cells play an important role in this process [29, 30, 34]; so, these contradictory data make it difficult to understand the role of Kupffer cells in liver fibrogenesis, which indicates the need for basic research.

At the early stages of liver fibrosis initiation and development (3^rd^ week of the experiment), an increase in the area of connective tissue was observed. At the same time, the *mmp-9 *mRNA level slightly decreased when compared to the control group. Probably, this decrease can be considered as one of the factors behind the relatively rapid accumulation of extracellular matrix proteins. The decreased *mmp-9 *mRNA level may be also associated with a general toxic effect in response to TAA exposure and more complex processes. First, fibrosis development is characterized by an imbalance between the production of metalloproteinases and the corresponding inhibitors (TIMP family proteins). It is likely that the inhibitors are induced before the cells begin to produce more metalloproteinases in response to toxic damage. Second, *mmp-9 *expression is controlled by epigenetic mechanisms and the increase in its expression may take some time and/ or be inhibited at the transcriptional level.

By the 5^th^ week of the experiment and in the presence of an increased *mmp*-9 mRNA level, the rate of connective tissue formation significantly decreased, which was probably due to the increased expression of the corresponding protein, leading to an effective destruction of collagen and other proteins of the extracellular matrix. Such a reaction can be considered an attempt by the organ to counteract fibrosis progression through metalloproteinase hyperactivation; so, a similar situation was observed at the 7^th^ week as well. In the presence of a slight increase in the *mmp-9* mRNA level when compared to the 5^th^ week, the rate of connective tissue synthesis continued to decrease. Hence, compared with the control group, the increase in the connective tissue area by the 3^rd^ week amounted to 2.1 times (201%, *p * < 0.05), 26.6% by the 5^th^ week (*p * < 0.05), and only 5.2% by the 7^th^ week (*p * < 0.05).

At the onset of the fibrosis to cirrhosis transition, the level of *mmp-9 *mRNA reached its maximum having increased 2.07 times (*p * < 0.05) against that of the control group. However, the growth of connective tissue increased markedly only by the 9th week to amount to approximately 50% (*p * < 0.05) when compared to its amount registered by the 7^th^ week. It seems that at this stage a role reversal takes place and the *mmp-9 *level ceases to be an important factor in curbing the development of fibrosis. It is possible that these are inflammation-associated factors that move to the fore, since their role noticeably increases, which is confirmed by the increased number of diffuse foci in the lymphoid-histiocytic infiltrate.

By the 11th week at the stage of incomplete cirrhosis, the *mmp-9 *mRNA level had decreased and probably ceased to play an important role in regulating the growth rate of the connective tissue area; so, its decrease was to do with some alternative protection/ regeneration mechanisms. The *mmp-9 *mRNA level increased only by 13% (*p* < 0.05) compared to the 9th week. At the same time, the signs of inflammation became more pronounced, significantly increasing the number of lymphoid-histiocytic infiltrate cells in the septa and portal zones.

At the stage of developed cirrhosis (weeks 13–17), the *mmp-9 *mRNA level reached that of the control group while the inflammation level in the liver reached its maximum.

Applying the immunohistochemical method has enabled us to simultaneously detect two morphologically heterogeneous myofibroblast populations in the liver of rats, which expressed different marker types. It is remarkable that in the early stages of fibrosis, the α-SMA+ cells were not lumped together with the FAP+ cells; before the onset of the fibrosis to cirrhosis transformation, α-SMA+ cells were noted in the sinusoids of the liver and necrosing foci only to be later localized both in the sinusoids and in the connective tissue septa. At the stage of portal fibrosis, the FAP+ cells were located near the interlobular vessels and interlobular bile ducts of the portal zones, and in the 7^th^ week, they were detected in the connective tissue septa and sinusoids.

The low level of inflammation before the fibrosis to cirrhosis transition suggests that the function of the cells producing the CD45 marker is to participate in the regulation of the functions of the polymorphic cells of pathological septa. But confirming this statement requires further and more detailed research.

## CONCLUSION

The results of our study have demonstrated that, when investigating liver fibrogenesis, the choice of an optimal reference gene depends on the fibrosis stage. If one is studying its specific stages and not the entire process in a long-term experiment, the optimal reference gene should be selected additionally, while the* mmp-9 *mRNA expression level should be considered as a marker for liver fibrosis development initiation and not as that for cirrhosis progression.

Applying the immunohistochemical method has enabled us to simultaneously uncover two morphologically heterogeneous myofibroblast populations that synthesize different marker types. The FAP+ cells have been found to be the main contributor to the development of the portal and initial stages of bridging fibrosis. They can be considered as one of the myofibroblast populations in thioacetamide-induced liver fibrogenesis. In the selected experimental model, fibrosis initiation and development before the start of parenchymal restructuring has proceeded at a low inflammation level.

## Abbreviations

HSCs: stellate cells
PFs: portal fibroblasts
TAA: thioacetamide
RT-PCR: real-time polymerase chain reaction

## References

[1] Tsomidis I., Notas G., Xidakis C., Voumvouraki A., Samonakis D.N., Koulentaki M., Kouroumalis E. (2022). Biomedicines..

[2] Rezaeian A.A., Yaghobi R., Geramizadeh B. (2018). Trop. Biomed..

[3] Wanninger J., Walter R., Bauer S., Eisinger K., Schäffler A., Dorn C., Weiss T.S., Hellerbrand C., Buechler C. (2011). Mol. Pathol..

[4] Lachowski D., Cortes E., Rice A., Pinato D., Rombouts K., Del Rio Hernandez A. (2019). Sci. Rep..

[5] Roeb E. (2018). Matrix. Biol..

[6] Boeker K.H.W., Haberkorn C.I., Michels D., Flemming P., Manns M.P., Lichtinghagen R. (2022). Clin. Chim. Acta..

[7] Craig V.J., Zhang L., Hagood J.S., Owen C.A. (2015). Am. J. Respir. Cell. Mol. Biol..

[8] Lu L., Zhang Q., Wu K., Chen X., Zheng Y., Zhu C., Wu J. (2015). Cancer Lett..

[9] Crespo I., San-Miguel B., Fernández A., de Urbina J.O., González-Gallego J., Tuñón M.J. (2015). Transl. Res..

[10] Su F., Zhang W., Chen Y., Ma L., Zhang H., Wang F. (2014). Exp. Ther. Med..

[11] Gadd V.L., Melino M., Roy S., Horsfall L., O’Rourke P., Williams M.R., Irvine K.M., Sweet M.J., Jonsson J.R., Clouston A.D., Powell E.E. (2013). Liver Int..

[12] Luo N., Li J., Wei Y., Lu J., Dong R. (2021). Physiol. Res..

[13] Baglieri J., Brenner D.A., Kisseleva T. (2019). Int. J. Mol. Sci..

[14] Lay A.J., Zhang H.E., McCaughan G.W., Gorrell M.D. (2019). Front. Biosci. (Landmark Ed)..

[15] Dhar D., Baglieri J., Kisseleva T., Brenner D.A. (2020). Exp. Biol. Med. (Maywood)..

[16] Fuji H., Miller G., Nishio T., Koyama Y., Lam K., Zhang V., Loomba R., Brenner D., Kisseleva T. (2021). Front. Mol. Biosci..

[17] Wells R.G. (2014). Curr. Pathobiol. Rep..

[18] Sun Y., Liu B., Xie J., Jiang X., Xiao B., Hu X., Xiang J. (2022). Mol. Med. Rep..

[19] Lebedeva E.I., Shchastniy A.T., Babenka A.S. (2022). Molecular medicine..

[20] Korzhevsky D.E. (2014). Theoretical foundations and practical application of immunohistochemistry method St. Petersburg: SpecLit, 2014. 119p..

[21] Zheng C., Luo J., Yang Y., Dong R., Yu F.X., Zheng S. (2021). Front Pediatr..

[22] Everhart J.E., Wright E.C., Goodman Z.D., Dienstag J.L., Hoefs J.C., Kleiner D.E., Ghany M.G., Mills A.S., Nash S.R., Govindarajan S. (2010). Hepatology..

[23] Lebedeva E.I., Shchastniy A.T., Krasochko P.A., Babenka A.S. (2022). Veterinary Journal of Belarus..

[24] Zhizhin K.S. (2007). Medical statistics: Textbook. Rostov n/a: Phoenix, 2007. 160p..

[25] Bustin S. (2004). A-Z of Quantitative PCR. La Jolla: International University Line, 2004. 882 p..

[26] Livak K.J., Schmittgen T.D. (2001). Methods..

[27] Mirzavand S., Rafiei A., Teimoori A., Khorsandi L., Bahreini A., Motamedfar A., Beiromvand M. (2020). Parasitol. Res..

[28] Ebrahim H.A., Kamar S.S., Haidara M.A., Abdel Latif N.S., Abd Ellatif M., ShamsEldeen A.M., Al-Ani B., Dawood A.F. (2022). Naunyn Schmiedebergs Arch. Pharmacol..

[29] Tacke F., Trautwein C. (2015). J. Hepatol..

[30] Murphy F.R., Issa R., Zhou X., Ratnarajah S., Nagase H., Arthur M.J.P., Benyon C., Iredale J.P. (2002). J. Biol. Chem..

[31] Wang Q., Liu X., Zhang J., Lu L., Feng M., Wang J. (2019). Mol. Med. Rep..

[32] Kobayashi T., Kim H., Liu X., Sugiura H., Kohyama T., Fang Q., Wen F., Abe S., Wang X. (2014). Am J. Physiol. Lung. Cell. Mol. Physiol..

[33] Lo R.C., Kim H. (2017). Clin. Mol. Hepatol..

[34] Atta H., El-Rehany M., Hammam O., Abdel-Ghany H., Ramzy M., Roderfeld M., Roeb E., Al-Hendy A., Abdel Raheim S., Allam H., Marey H. (2014). PLoS One..

